# Supplementary material for: Gut microbiota metabolite tyramine ameliorates high-fat diet-induced insulin resistance via increased Ca2+ signaling
Source: EMBO J. 2024 Jul 4;43(16):3466–93. doi: 10.1038/s44318-024-00162-w (PMC11329785; doi:10.1038/s44318-024-00162-w)
Supplement: Supplementary file 1 — Appendix [file 44318_2024_162_MOESM1_ESM.pdf]

Dysbiosis-produced tyramine ameliorates high-fat diet-induced insulin resistance via Ca^{2+} signaling

Peng Ma, Yao Zhang, Youjie Yin, Saifei Wang, Shuxin Chen, Xueping Liang, Zhifang Li and Hansong Deng*

*: hdeng@tongji.edu.cn

Yangzhi Rehabilitation Hospital, Sunshine Rehabilitation Center, Frontier Science Center for Stem Cell Research, School of Life Sciences and Technology, Tongji University, Shanghai 20092, China.

Table of contents: 4 supplemental figures in the appendix

Appendix Figure 1……………………………………1-3

Appendix Figure 2……………………………………3-4

Appendix Figure 3……………………………………5-6

Appendix Figure 4……………………………………6

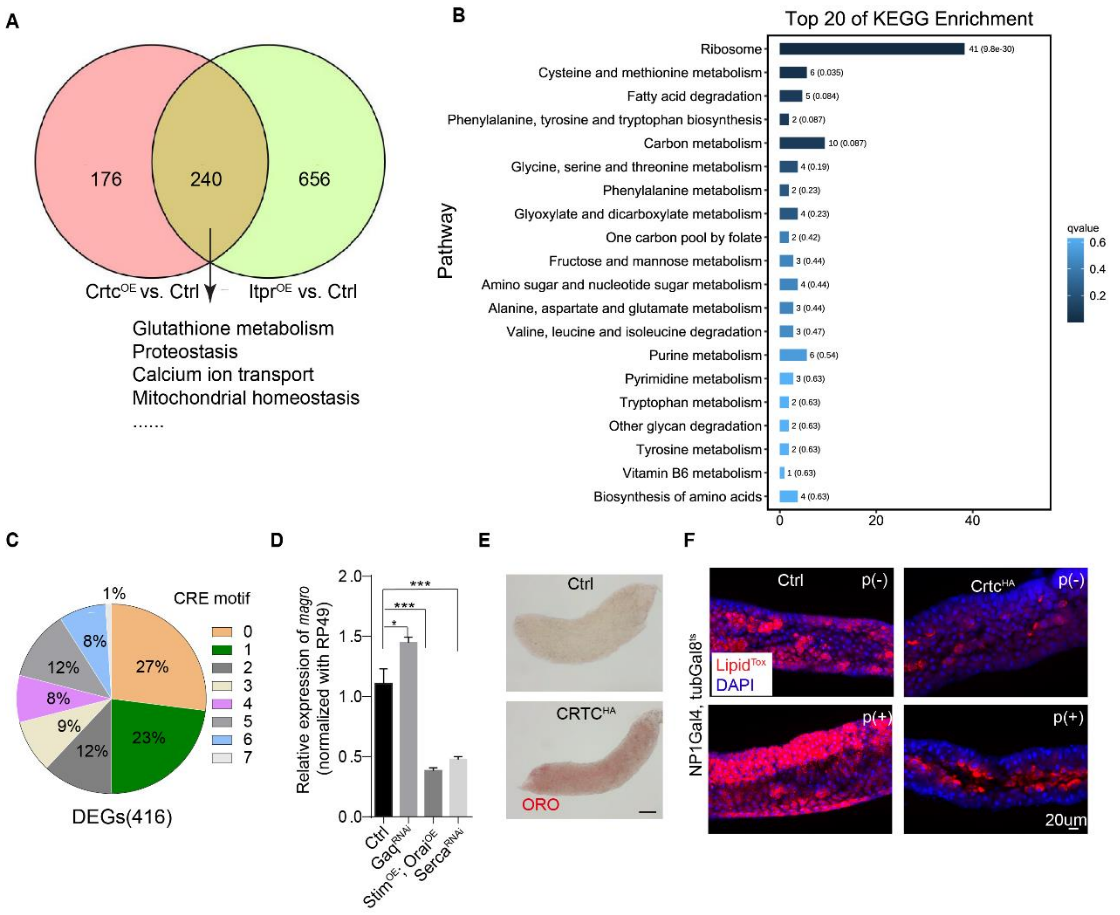

Appendix Figure S1. related to **Fig.5 Ca^{2+}/CRTC/CREB cascade inhibits dietary lipid uptake by suppressing transcription of *magro.***

A, Venn diagram comparing DEGs regulated by CRTC overexpression or IP3R overexpression. Genes involved in glutathione metabolism, proteostasis, Ca^{2+} ion transport, and mitochondrial homeostasis are enriched among the common genes, which are known processes regulated by the CRTC/CREB module.

B, The DEGs were plotted into gene pathways using the KEGG pathway enrichment analysis. Each bar represents the total number of genes in the pathway. Diverse colors indicate different q value.

C, The percentage of CRE containing DEGs(416) in the promoter region (up to 2kb upstream of TSS) was shown by bar chart. The number of CREs was displayed in different colors.

D, Expression of *magro* in gut was quantified by qRT-PCR. Two-way ANOVA analysis for statistics. *: $p< 0.05$, ***: $p<0.001$. Three independent experiments were performed. mean± S.E.M shown. Genotypes: NP1Gal4, tubGal80^{ts}; UAS-*Serca*^{RNAi} or NP1Gal4, tubGal80^{ts}; UAS-*Gaq*^{RNAi} or NP1Gal4, tubGal80^{ts}; UAS-*Stim*^{OE}*, UAS-Orai*^{OE}.

E, Related to **Fig.5F,** Lipid levels in fecal samples were increased by CRTC overexpression. Representative images were shown. Scale bar: 100μm.

F, Related to **Fig.5G**, Representative images showed that flies fed with pancreatin-containing food (5mg/ml, 4d) significantly restored lipid levels reduced by CRTC overexpression. LipidTOX stains neutral lipids in red. Scale bars: 20μm. Genotypes for

E and F: Genotype: NP1Gal4, tubGal80^{ts} or NP1Gal4, tubGal80^{ts}; UAS-*CRTC^{HA}*.

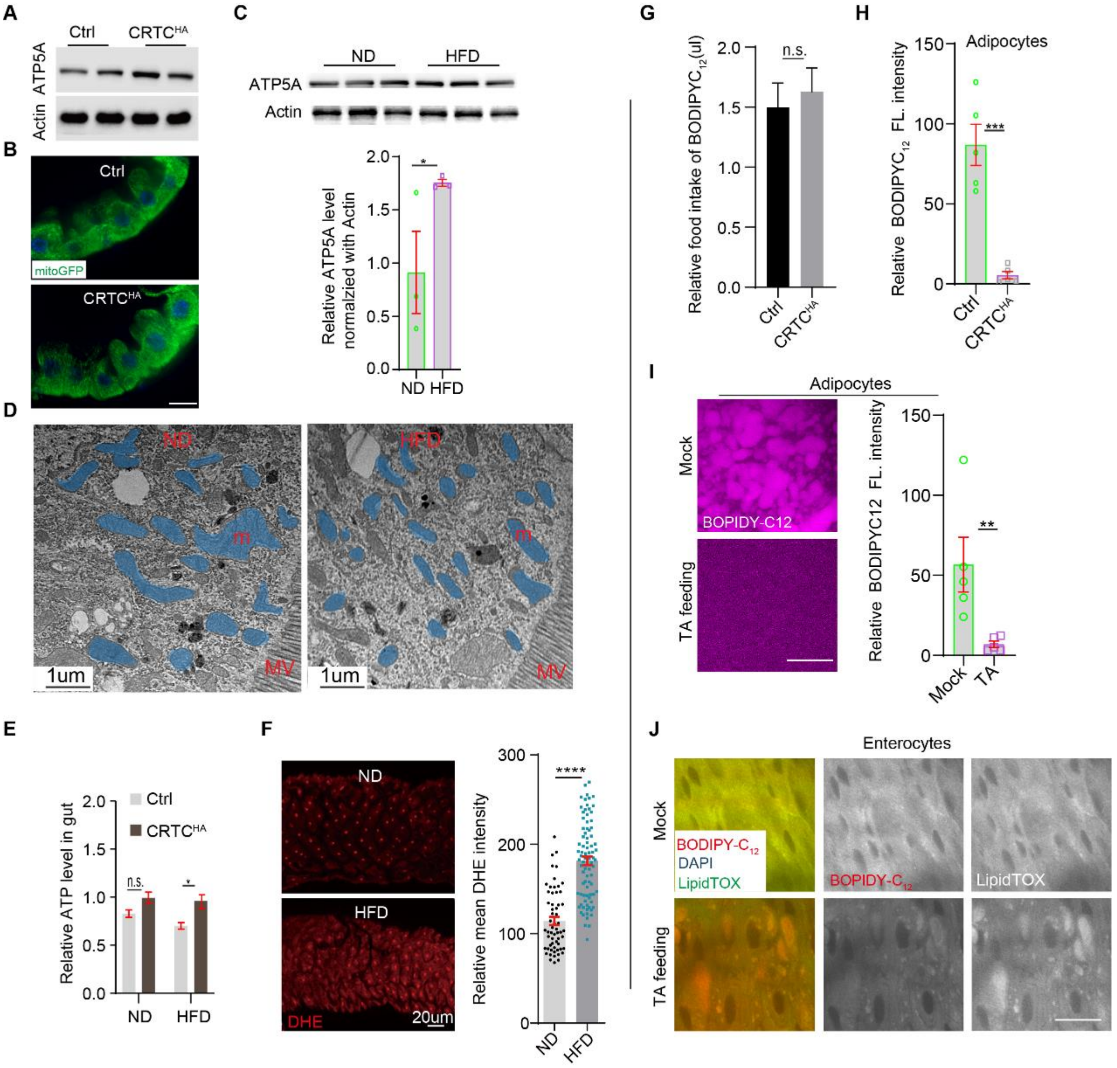

Appendix Figure S2. Related to **Fig.6 The Ca^{2+}/CRTC/CREB cascade promotes mitochondrial biogenesis and inhibits lipogenesis in enterocytes.**

A, Related to Fig. 6C, representative images of Western blot showing that ATP5A protein level in the intestine was increased by CRTC overexpression.

B, Representative confocal images of cross-sectioned gut samples showing that mitochondrial mass in ECs was increased by CRTC overexpression. mitoGFP: mitochondrial-targeted GFP. Genotype: NP1Gal4, tubGal80^{ts}; UAS::mito-GFP, UAS-*CRTC^{HA}*. Scale bar: 10μm.

C, Western blot images showing that ATP5A protein level in the intestine was increased by HFD. Quantifications shown in the bottom. Three independent experiments were performed, t-Test for statistics, mean± S.E.M were shown. *: p<0.05.

D, Representative TEM images showing that mitochondrial mass in ECs was increased under HFD condition. Typical mitochondria were pseudo-colored in blue, MV: microvilli, m: mitochondria, scale bar: 1μm.

E, Relative ATP level in guts were quantified. Three independent experiments were performed. Mean ± S.E.M. were shown. *: $p<0.05$, n.s.: no significance.

F, HFD feeding significantly increases ROS levels (DHE staining, red) in ECs. A representative image is shown on the left, and quantification shown on the right. Approximately 80 ECs from 6 guts of each condition. t-Test for statistics, mean± S.E.M were shown. ****: $p<0.0001$. Scale bars: 20μm.

G, Relative food intake of BODIPY-C_{12} was measure by CAFÉ assay with food colored dye. Biological triplicates were performed 10 animals per group. t-Test for statistics. Mean ± S.E.M. are shown. n.s.: no significance.

H, **Related to Fig.6F**, relative BODIPY-C_{12} fluorescence intensity in adipocytes of animals with gut specific CRTC overexpression were quantified. or TA feeding(I)was shown and quantified. Three independent experiments were performed. n=6 for each condition. t-Test for statistics. Mean ± S.E.M. are shown. ***: $p<0.001$.

I, BODIPY-C_{12} fluorescence intensity in adipocytes of animals fed with TA. Representative images shown on the left, quantifications on the right. Three independent experiments were performed. n=6 animals for each condition. t-Test for statistics. Mean ± S.E.M. are shown. **: $p<0.01$. Scale bar: 20μm.

J, Distribution of BODIPY-C_{12} in ECs after TA feeding (1mg/ml, 24hr) was examined by Spinning Disk Confocal Super Resolution (spinSR) microscopy. Representative images shown. LipidTOX stains neutral lipids(green), BODIPY-C_{12}(red). Scale bar: 20μm.

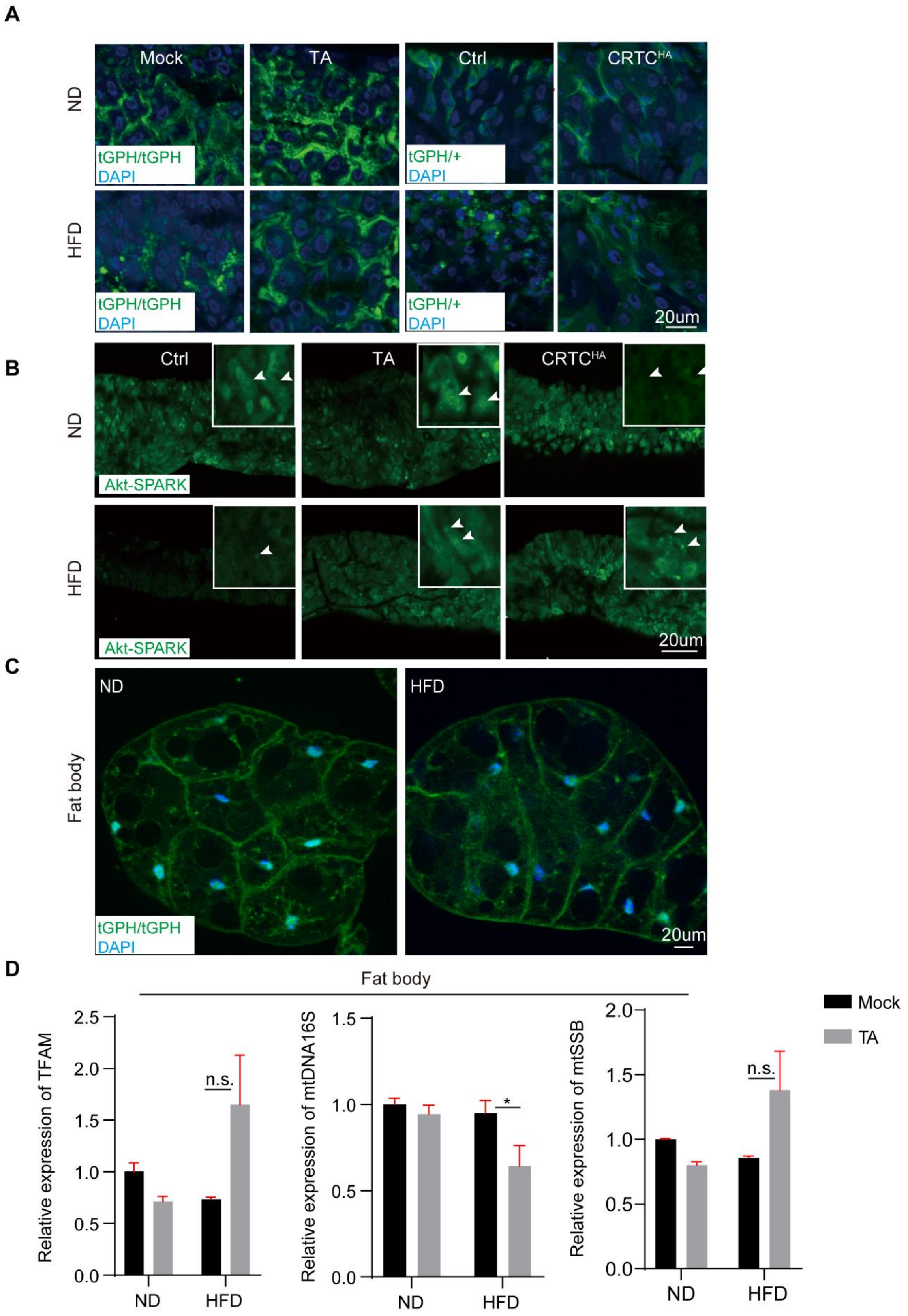

Appendix Figure S3. Related to **Fig. 7 CRTC/CREB cascade suppresses HFD-mediated insulin resistance.**

A, Chronic HFD feeding significantly suppresses membrane-bound t–GPH signals (green). TA feeding or CRTC overexpression in ECs can partially restore membrane-

bound t-GPH. Representative images are shown. Scale bars: 20μm.

B, related to Fig. **7A**. Chronic HFD feeding (14 d) significantly suppresses AKT activity (indicated by Akt-SPARK signals), and feeding with TA or CRTC overexpression in ECs partially restore Akt-SPARK droplets. Representative images are shown, and insets are higher mag images of the boxed area. White arrowheads point to typical AMPK-SPARK positive puncta. Scale bar: 20μm.

C, Membrane-bound t-GPH (green) in fat body was examined under HFD condition. Representative images are shown. Nuclei counterstain in blue by DAPI. Scale bar: 20μm.

D, Expression of TFAM, mtDNA16S, and mtSSB in fat body was examined by RT-q-PCR under indicated conditions. Triplicates were performed, mean ± S.E.M. were shown. t-Test for statistics. *: p<0.05, n.s.: no significance.

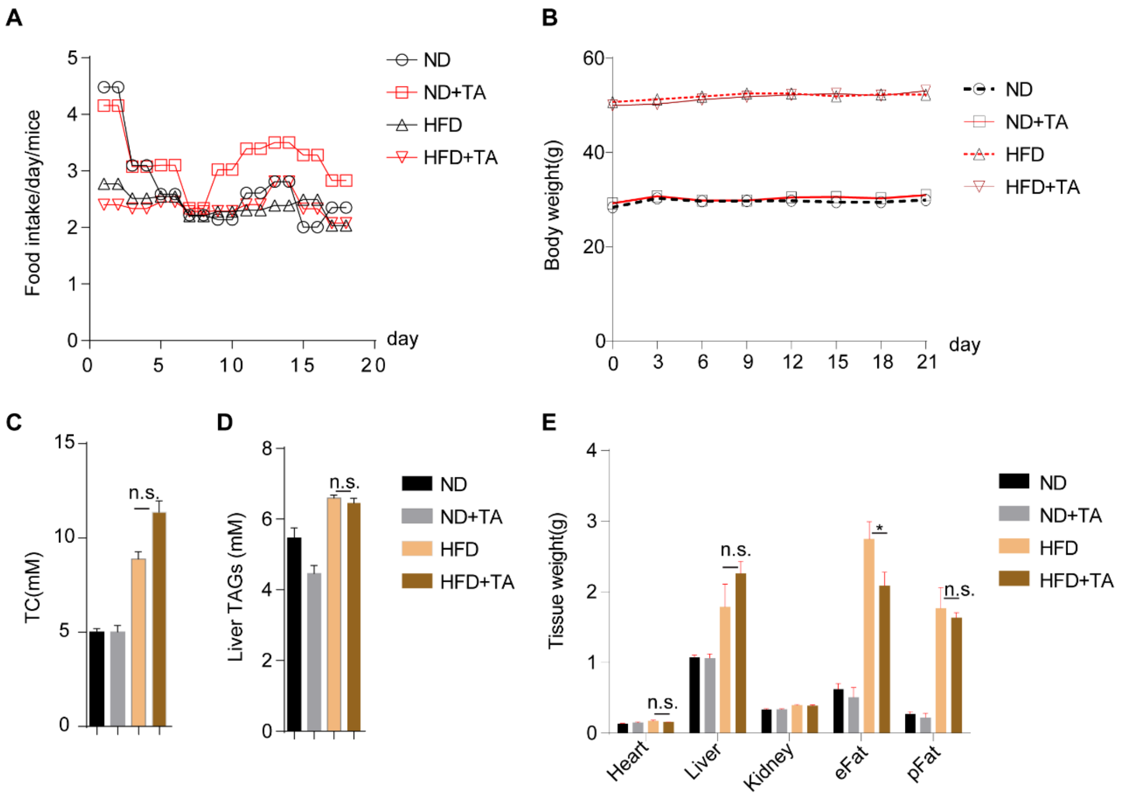

Appendix Figure S4. Related Fig.9 **TA alleviates HFD-induced insulin resistance in mice**

A-B, Food intake and body weight was not reduced by TA ingestion (0.04%).

C-D, Total cholesterol (TC) and TAG levels in the liver of ND or HFD mice were quantified after TA administration.

E, Tissue mass in mice fed with ND or HFD after TA ingestion. Data shown represent the Mean± S.E.M. n=6 per group. Student t-test for statistics. *: p<0.05, n.s.: no significance.
