# Supplementary material for: Gut microbiota metabolite tyramine ameliorates high-fat diet-induced insulin resistance via increased Ca2+ signaling
Source: EMBO J. 2024 Jul 4;43(16):3466–93. doi: 10.1038/s44318-024-00162-w (PMC11329785; doi:10.1038/s44318-024-00162-w)
Supplement: Supplementary file 3 — Movie EV1 [file 44318_2024_162_MOESM3_ESM.zip › Movie_EV1_legend.docx]

**Movie EV1:** No obvious oscillations of cytosolic calcium were observed in enterocytes of fly gut by two-photon live imaging. Genotype: 5966^GS^GAL4; *UAS-tdTomato-P2A-GCaMP5G*
