# Supplementary material for: Gut microbiota metabolite tyramine ameliorates high-fat diet-induced insulin resistance via increased Ca2+ signaling
Source: EMBO J. 2024 Jul 4;43(16):3466–93. doi: 10.1038/s44318-024-00162-w (PMC11329785; doi:10.1038/s44318-024-00162-w)
Supplement: Supplementary file 13 — Expanded View Figures [file 44318_2024_162_MOESM13_ESM.pdf]

Expanded View Figures

Figure EV1. Related to Fig. 1, cytoCa^{2+} in enterocytes was activated by HFD to reduce lipid levels through the Gαq/PLCβ/IP3R cascade.

(**A**) CytoCa^{2+} levels in ECs of the R2 region. Subcellular localization of Ca^{2+} signals (GCaMP3, green), and F-actin (LifeactRFP, red) in ECs of the R2 region were shown under confocal microscopy. Insets are high-mag images of the boxed area. Top: Sagittally view, bottom superficially view. Scale bars: 10 μm. Genotype: UAS-GCaMP3; NP1Gal4^{ts}; UAS-LifeactRFP. (**B**) Distribution of neutral lipid levels (Lipid^{TOX}, red) and cytoCa^{2+} levels (GCaMP3, green) were examined in the R2 region. Representative images are shown. Scale bars: 50μm. Genotypes: UAS-GCaMP3; NP1Gal4, tubGal80^{ts}; UAS-*Serca*^{RNAi} or UAS-GCaMP3; NP1Gal4, tubGal80^{ts}; UAS-*IP3R*^{RNAi}. (**C**, **D**) Overexpressing Stim and Orai in ECs is sufficient to reduce lipid content in the gut and whole body. Neutral lipid levels were measured by a TAG kit. Triplicates were performed for statistical purposes. *t* test for statistics, mean ± SEM is shown. *$P < 0.05$. Genotype: UAS-GCaMP3; NP1Gal4, tubGal80^{ts}; UAS-Stim, UAS-Orai. (**E**) Related to Fig. 1G, Intestinal neutral lipids were increased by coconut oil (30%) containing HFD. Representative images were shown. Scale bar: 100 μm. (**F**) Intestinal neutral lipids were also increased by lard oil (30%) containing HFD. ORO intensity in the R2 region was quantified. Three independent experiments were performed. $n >= 10$ animals for each experiment. Mean ± SEM were shown, *t* test for statistics. **$P < 0.01$. (**G**) cytoCa^{2+} in ECs was recorded after feeding with lard oil containing HFD for 4 days. cytoCa^{2+} was quantified as relative fluorescence ratio of GCaMP5 vs. tdTomato. At least 80 enterocytes from 6 guts were quantified. Each dot represents one EC. *t* Test for statistics. ****$P < 0.0001$. Genotype: NP1Gal4, tubGal80^{ts}; *UAS-tdTomato-P2A-GCaMP5G*. (**H**, **I**) Knockdown of *IP3R* (**H**) or *Gaq* (**I**) in ECs increased ORO intensities in guts under both ND and HFD conditions. Three independent experiments were performed. $n = 6$ animals for each experiment. Two-way ANOVA analysis for (**H**), *t* test for (**I**). Mean ± SEM were shown., *$P < 0.05$, **$P < 0.01$, ***$P < 0.001$.

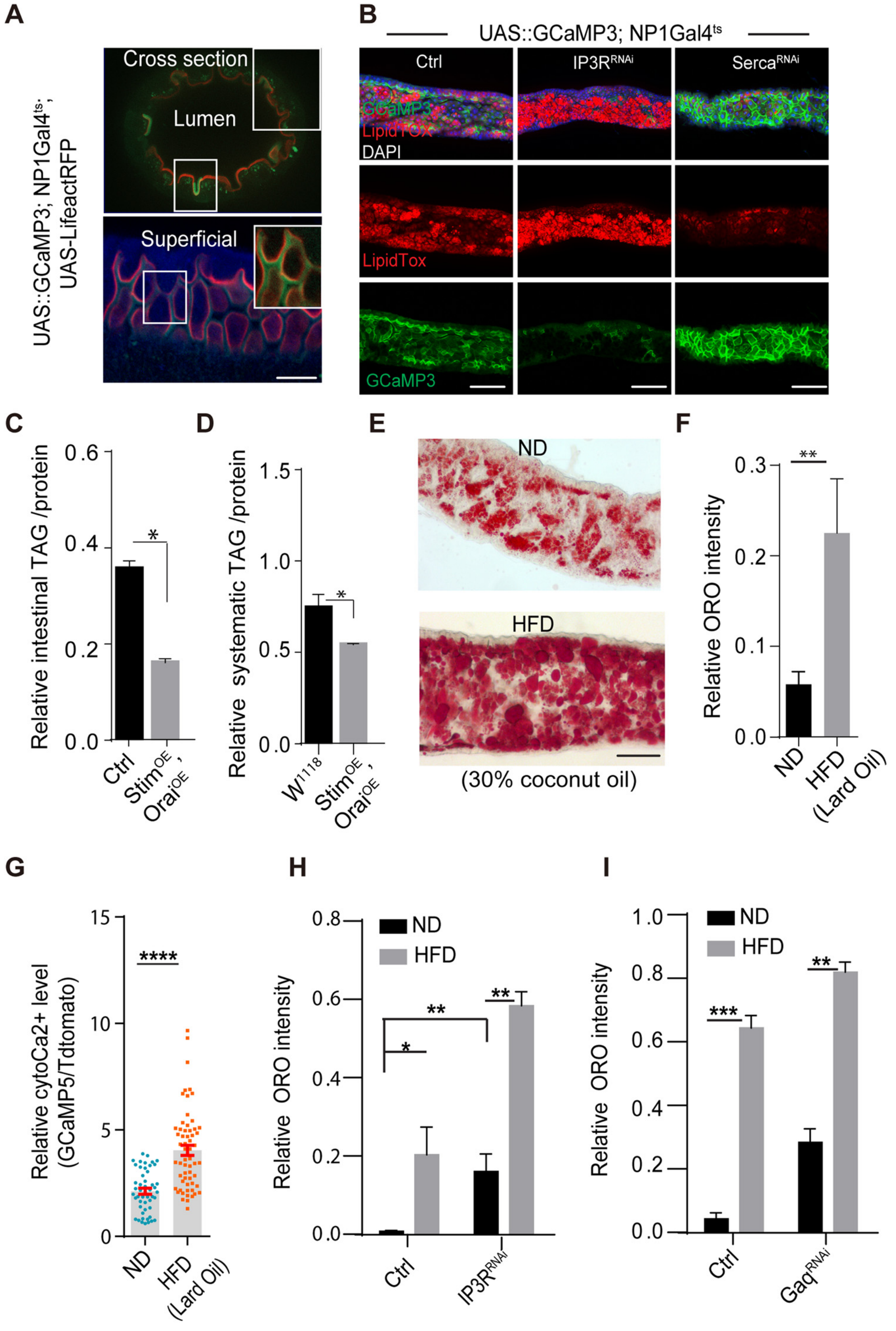
A
UAS::GCaMP3; NP1Gal4ts; UAS-LifeactRFP
Cross section
Lumen
Superficial
B
UAS::GCaMP3; NP1Gal4ts
Ctrl
IP3RRNAi
SercaRNAi
GCaMP3
LipidTox
DAPI
LipidTox
GCaMP3
C
Relative intestinal TAG /protein
0.6
0.4
0.2
0.0
*
Ctrl
StimOE, OraiOE
D
Relative systematic TAG /protein
1.5
1.0
0.5
0.0
*
W1118
StimOE, OraiOE
E
ND
HFD
(30% coconut oil)
F
Relative ORO intensity
0.3
0.2
0.1
0.0
**
ND
HFD (Lard Oil)
G
Relative cytoCa2+ level (GCaMP5/Tdtomato)
15
10
5
0
****
ND
HFD (Lard Oil)
H
Relative ORO intensity
0.8
0.6
0.4
0.2
0.0
ND
HFD
*
**
**
Ctrl
IP3RRNAi
I
Relative ORO intensity
1.0
0.8
0.6
0.4
0.2
0.0
ND
HFD
***
**
Ctrl
GaqRNAi

 © The Author(s)

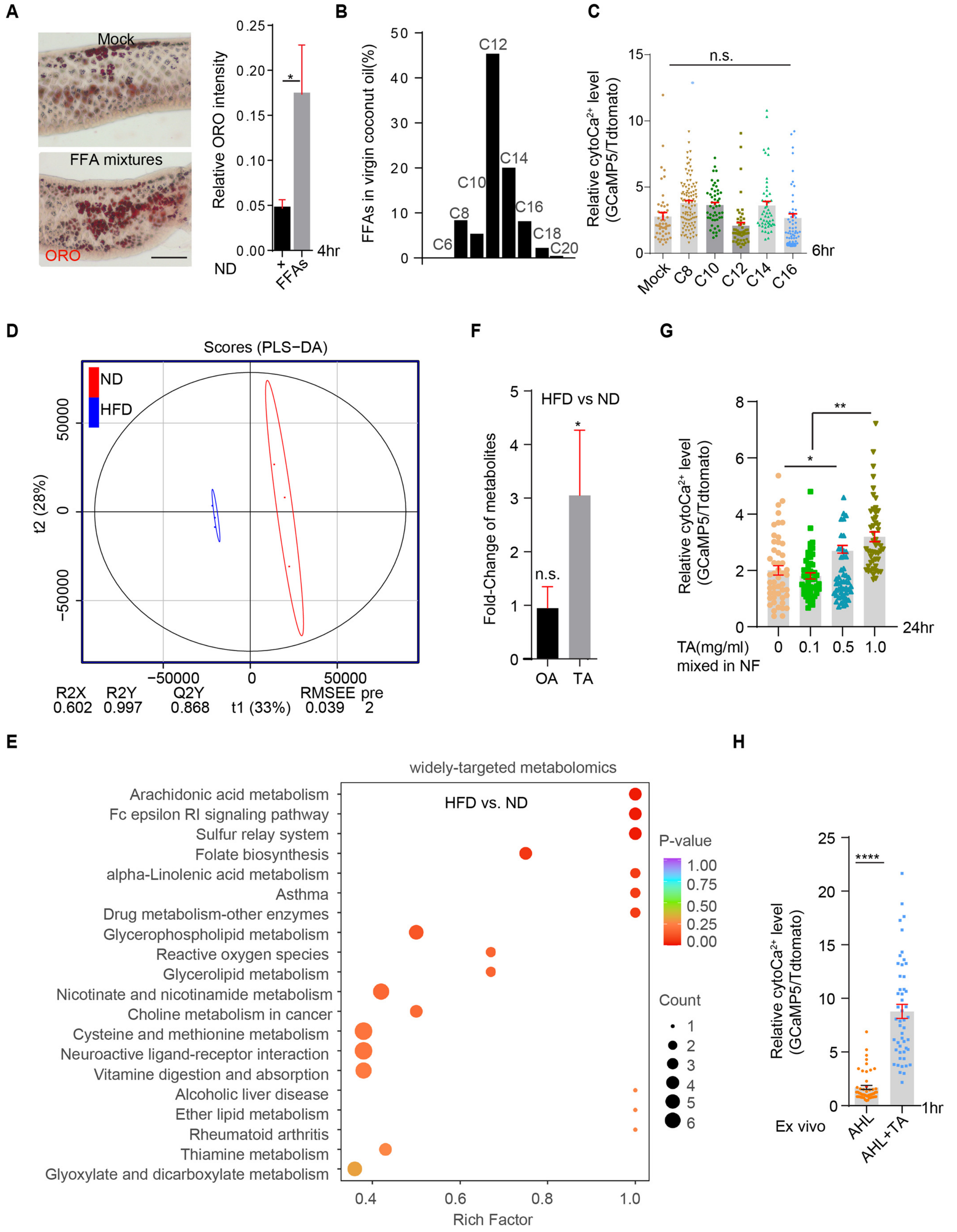
A
Mock
FFA mixtures
ORO
Relative ORO intensity
ND
FFAs
4hr
B
FFAs in virgin coconut oil(%)
C6 C8 C10 C12 C14 C16 C18 C20
C
n.s.
Relative cytoCa2+ level (GCaMP5/Tdtomato)
Mock C8 C10 C12 C14 C16
6hr
D
Scores (PLS−DA)
ND
HFD
t2 (28%)
t1 (33%)
R2X R2Y Q2Y RMSEE pre
0.602 0.997 0.868 0.039 2
F
HFD vs ND
Fold-Change of metabolites
n.s.
OA TA
G
Relative cytoCa2+ level (GCaMP5/Tdtomato)
TA(mg/ml) mixed in NF
24hr
E
widely-targeted metabolomics
HFD vs. ND
Arachidonic acid metabolism
Fc epsilon RI signaling pathway
Sulfur relay system
Folate biosynthesis
alpha-Linolenic acid metabolism
Asthma
Drug metabolism-other enzymes
Glycerophospholipid metabolism
Reactive oxygen species
Glycerolipid metabolism
Nicotinate and nicotinamide metabolism
Choline metabolism in cancer
Cysteine and methionine metabolism
Neuroactive ligand-receptor interaction
Vitamine digestion and absorption
Alcoholic liver disease
Ether lipid metabolism
Rheumatoid arthritis
Thiamine metabolism
Glyoxylate and dicarboxylate metabolism
Rich Factor
P-value
Count
H
Relative cytoCa2+ level (GCaMP5/Tdtomato)
Ex vivo
AHL
AHL+TA
1hr

Figure EV2. Related to Fig. 2: Tyramine induced by HFD promotes cytoCa^{2+} levels in enterocytes.

(**A**) Intestinal lipid levels were increased when flies were fed ND supplemented with mixtures of fatty acids derived from coconut oil for 4 h. Representative images of ORO staining are shown on the left, and quantifications on the right. Three independent experiments were performed. $n = 6$ animals for each condition. Mean ± SEM were shown. *t* test for analysis. $^*P < 0.05$ Scale bar: 200 μm. (**B**) Medium-chain fatty acid composition of virgin coconut oil. Modified from an earlier publication (Sacks, 2020). (**C**) Flies fed with ND supplemented with the indicated saturated fatty acids for 6 h failed to increase enteric cytoCa^{2+}. At least 80 enterocytes from 6 guts were quantified. *t* Test for analysis. Mean ± SEM were shown. n.s.: no significance. Genotype: NP1Gal4, tubGal80^{ts}; *UAS-tdTomato-P2A-GCaMP5G*. (**D**) The supervised multivariate method of partial least squares-discriminant analysis (PLS-DA) was used to maximize the metabolome differences between sample pair. PLS-DA score plots generated from PLS-DA models in HFD and ND. R2X = 0.602, R2Y = 0.997, Q2Y = 0.868; RMSEE represent Root Mean Square Error of Estimation. The scores plot of PLS-DA modeling shows a different clustering tendency. (**E**) The vertical axis represents the enriched KEGG classification. The horizontal axis is the rich factor (rich factor ≤1), which represents the ratio of the number of differentially expressed proteins to those identified in the KEGG pathway. The size of the circular area represents the number of differentially expressed proteins, and the circular color represents the enrichment *P* value of the differentially expressed proteins under the KEGG classification. (**F**) Widely targeted metabolomics indicated that tyramine (TA) readings are significantly increased while octopamine (OA) readings are largely unchanged. $n = 3$, mean ± SEM. *t* test for statistics, n.s.: no significance, $^*P < 0.05$. (**G**) CytoCa^{2+} in ECs was examined after flies were fed diets supplemented with TA at the indicated concentrations. Two-way ANOVA analysis for statistics. mean± SEM is shown, $^*P < 0.05$, $^{**}P < 0.01$. At least 60 enterocytes from 4 guts were quantified. Genotype: NP1Gal4, tubGal80^{ts}; *UAS-tdTomato-P2A-GCaMP5G*. (**H**) CytoCa^{2+} level in ECs from freshly dissected guts was increased after incubation with AHL plus TA (1 mg/ml) for 1 h. Approximately 50 ECs from 4 intestines were analyzed. *t* test for statistics, mean ± SEM, $^{****}P < 0.0001$.

 © The Author(s)

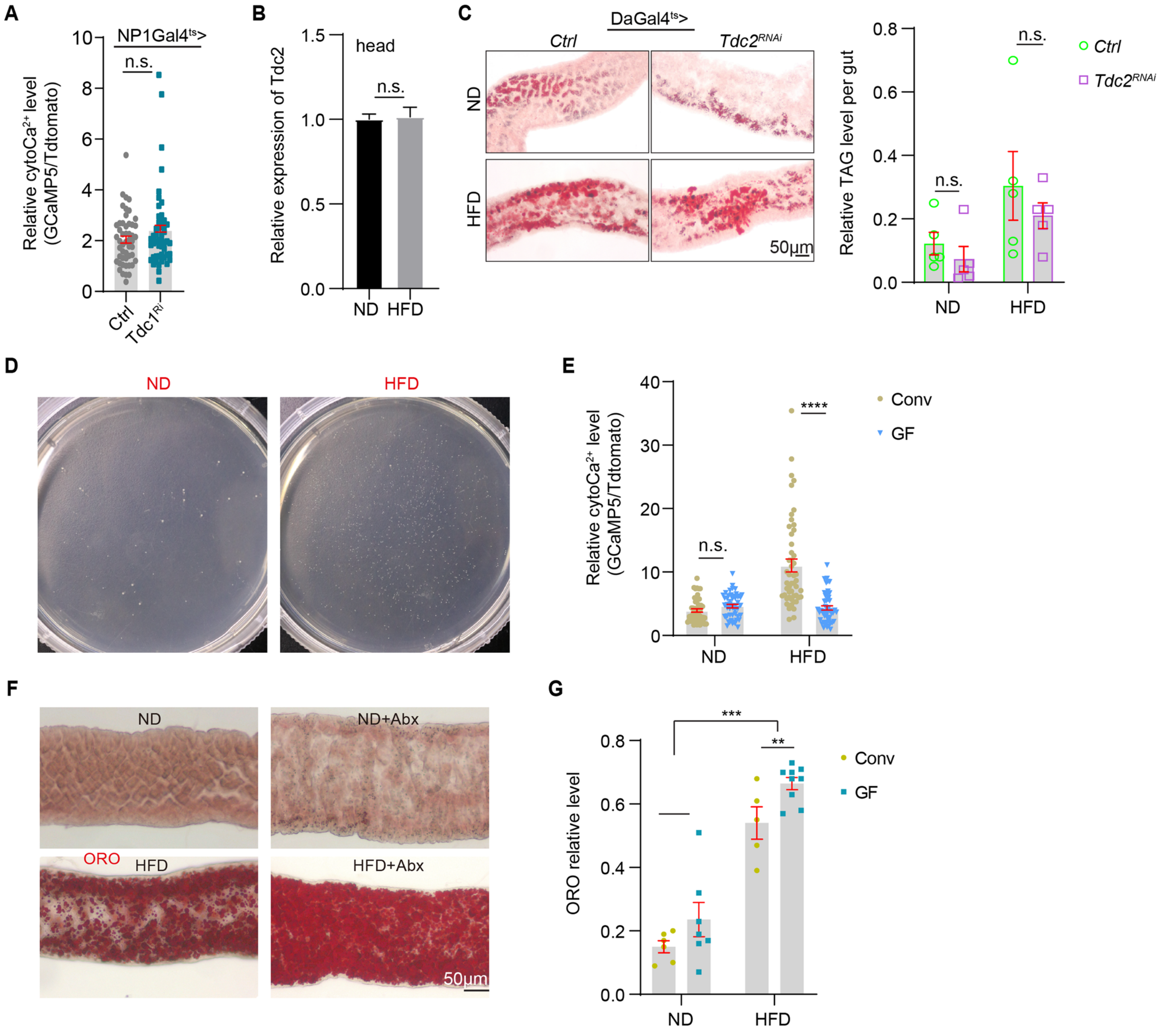

Figure EV3. Related to Fig. 3: Bacterial load in fly guts was increased by HFD.

(**A**) cytoCa2+ in ECs was examined when *tdc1* was temporally silenced in ECs by NP1Gal4. Approximately 50 ECs from 4 intestines were analyzed. *t* Test for statistics, n.s.: no significance, mean ± SEM. Genotype: NP1Gal4, tubGal80^{ts}; *UAS-tdTomato-P2A-GCaMP5G*. (**B**) *Tdc2* expression in fly head was examined by RT-qPCR. Means ± SEM from biological triplicates. Student *t* Test for statistics, n.s: no significance. (**C**) Lipid levels (indicated by ORO staining) in ECs were examined when *tdc2* was ubiquitously inhibited. Representative images shown on left and quantifications on the right. Biological triplicates performed for statistics. $n = 5$ for each condition. *t* test for statistics, mean ± SEM shown, n.s.: no significance. Scale bar: 50 μm. Genotype: DaGal4, tubGal80^{ts}; *UAS-tdc2^{RNAi}*. (**D**) HFD increases gut bacterial load. 50 μl of homogenized sample from gut lysates of 6 animals were plated onto each antibiotic-free nutrition agar plate. (**E**) Ca^{2+} levels in ECs are also significantly reduced in germ-free flies under HFD conditions. Relative cytoCa^{2+} levels in ECs were monitored by two-photon live imaging. Each dot represents one EC, at least 100 ECs from 6 guts were quantified for each condition. n.s.: no significance, ***$P < 0.001$. (**F**) Related to Fig. 3C, antibiotic cocktail (Abx) treatment further increases neutral lipid content in the intestine. Representative images of ORO staining in the R2 region are shown. Scale bar: 50 μm. (**G**) Germ-free (GF) flies contains more intestinal lipid content under HFD conditions than those raised under conventional condition (Conv). Lipid levels were examined by ORO staining. Biological triplicates performed. 6–8 animals analyzed for each condition. *t* test for statistics, mean ± SEM shown. **$P < 0.01$, ***$P < 0.001$.

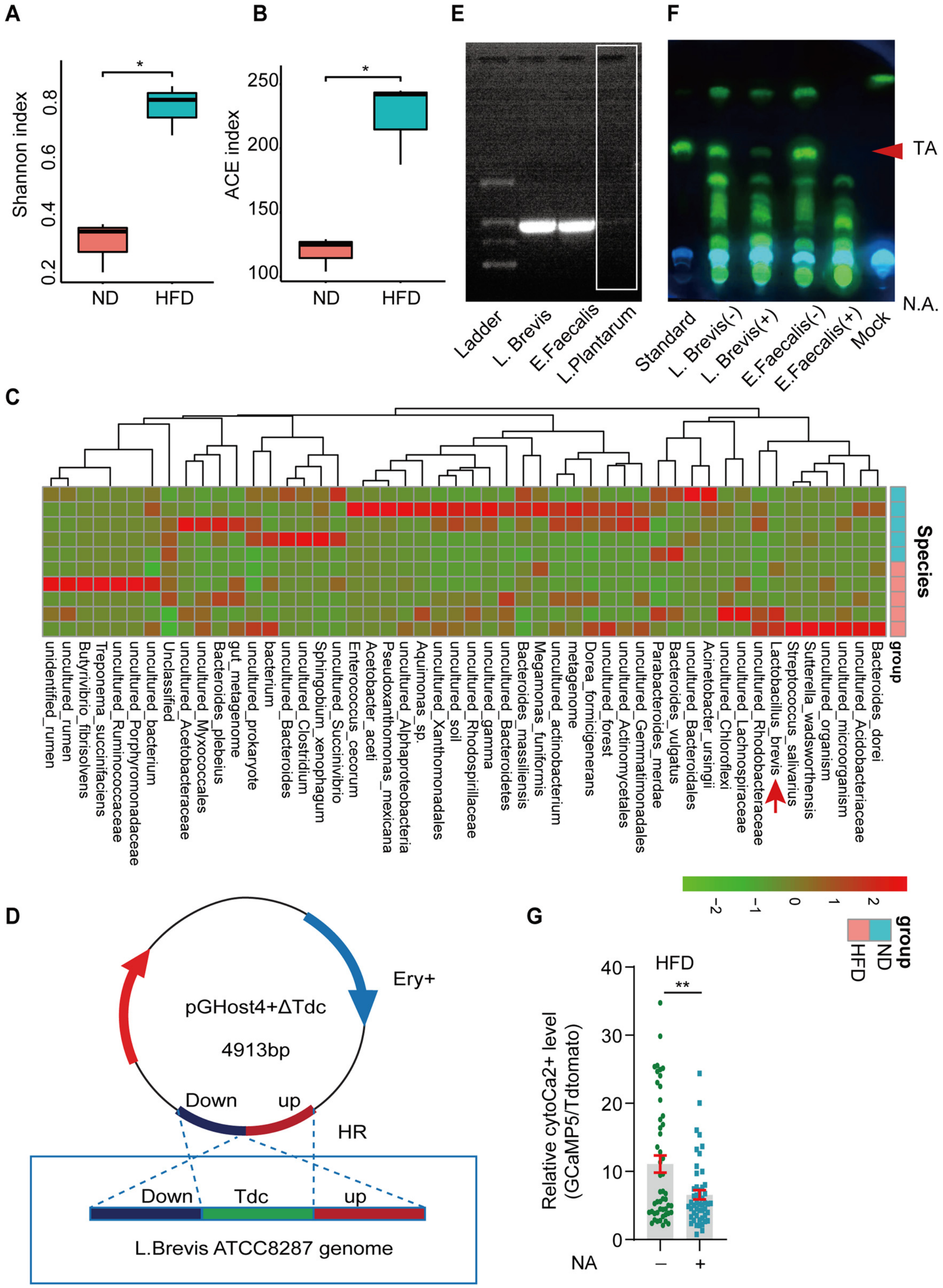
A
Shannon index
ND
HFD
B
ACE index
ND
HFD
E
Ladder
L. Brevis
E.Faecalis
L.Plantarum
F
TA
N.A.
Standard
L. Brevis(-)
L. Brevis(+)
E.Faecalis(-)
E.Faecalis(+)
Mock
C
Species
group
unidentified_rumen
uncultured_rumen
Butyrivibrio_fibrisolvens
Treponema_succinifaciens
uncultured_Ruminococcaceae
uncultured_Porphyromonadaceae
uncultured_bacterium
Unclassified
uncultured_Acetobacteraceae
uncultured_Myxococcales
Bacteroides_plebeius
gut_metagenome
uncultured_prokaryote
bacterium
uncultured_Bacteroides
uncultured_Clostridium
Sphingobium_xenophagum
uncultured_Succinivibrio
Enterococcus_cecorum
Acetobacter_aceti
Pseudoxanthomonas_mexicana
uncultured_Alphaproteobacteria
Aquimonas_sp.
uncultured_Xanthomonadales
uncultured_soil
uncultured_Rhodospirillaceae
uncultured_gamma
uncultured_Bacteroidetes
Bacteroides_massiliensis
Megamonas_funiformis
uncultured_actinobacterium
metagenome
Dorea_formicigenerans
uncultured_forest
uncultured_Actinomycetales
uncultured_Gemmatimonadales
Parabacteroides_merdae
Bacteroides_vulgatus
uncultured_Bacteroidales
Acinetobacter_ursingii
uncultured_Chloroflexi
uncultured_Lachnospiraceae
uncultured_Rhodobacteraceae
Lactobacillus_brevis
Streptococcus_salivarius
Sutterella_wadsworthensis
uncultured_organism
uncultured_microorganism
uncultured_Acidobacteriaceae
Bacteroides_dorei
-2
-1
0
1
2
group
ND
HFD
D
Ery+
pGHost4+ΔTdc
4913bp
Down
up
HR
Down
Tdc
up
L.Brevis ATCC8287 genome
G
HFD
**
Relative cytoCa2+ level
(GCaMP5/Tdtomato)
NA
–
+

© The Author(s)

Figure EV4. Related to Fig. 3, Gram-positive bacteria increased by HFD is responsible for tyramine production.

(**A**, **B**) The Shannon index and ACE index of Krushal–Wallis analysis of 16 S amplicon sequencing were used to plot species diversity and abundance respectively. *$P < 0.05$, $n = 5$. For Shannon index, The minima, maxima, center(median), and IQR(interquartile range) are marked in boxplots. (**C**) Heatmap of differentially expressed bacterial family in HFD v.s. ND was shown. The arrow points to the *Lactobacillaceae Brevis* (*L. Brevis*) species, which is significantly increased in the HFD condition. (**D**) Design for the generation of *tdc* deletion mutants in *L. Brevis* based on the homologous recombination (HR) strategy. For details, see "Methods". **(E)** Electrophoresis gel analysis from PCR using primer set for *tdc* gene. The product (941 bp in length) was not present in the *L. Plantarum* lane (the boxed one). (**F**) Thin-layer chromatography (TLC) showed that nicotine acid (N.A., 0.5 mg/ml) robustly reduced TA levels (red arrowhead) in the culture supernatant of *L. brevis* and *E. faecalis*. (**G**) The cytoCa^{2+} level in ECs was examined when flies were fed with N.A. (0.5 mg/ml) under HFD conditions. Approximately 40 ECs from 4 intestines were analyzed. *t* Test for statistics, **$P < 0.01$, mean ± SEM shown. Genotype: NP1Gal4, tubGal80^{ts}; *UAS-tdTomato-P2A-GCaMP5G*.

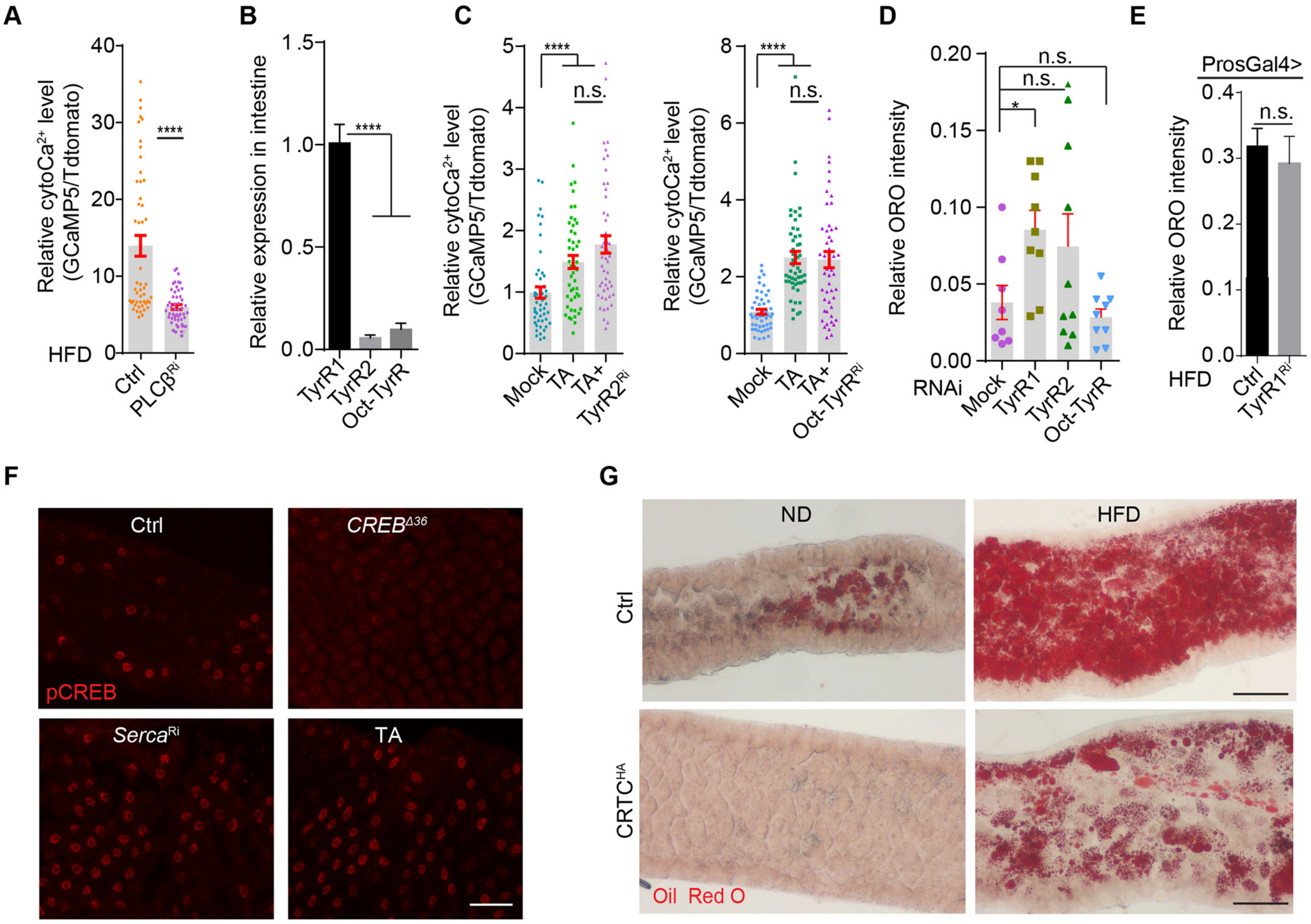

Figure EV5. Tyramine activates enteric Ca^{2+} signaling through tyramine receptor, *TyrR1*.

(**A**) PLCβ knockdown in ECs significantly reduces HFD-induced cytoCa^{2+} levels. Each dot denotes one EC, at least 60 ECs from 5 guts were quantified. Student *t* test for statistics. Mean ± SEM were shown. *****P* < 0.0001, Genotype: NP1Gal4, tubGal80^{ts}; *UAS-tdTomato-P2A-GCaMP5G, UAS-PLCβ*^{RNAi}. (**B**) Relative expression of tyramine receptors in fly guts by qRT-PCR. Means ± SEM from biological triplicates. Student *t* Test for statistics, *****P* < 0.0001. (**C**) Knockdown of *TyrR2* or *Oct-TyrR* in ECs failed to block TA-elevated cytoCa^{2+} levels. CytoCa^{2+} in ECs was recorded when freshly dissected guts were incubated with AHL containing TA(1 mg/ml). Biological triplicates were performed, mean ± SEM were shown. One-way ANOVA analysis for statistics, *****P* < 0.0001. n.s. no significance. Genotypes: NP1Gal4, tubGal80^{ts}; *UAS-tdTomato-P2A-GCaMP5G, UAS-Oct-TyrR*^{RNAi} or NP1Gal4, tubGal80^{ts}; *UAS-tdTomato-P2A-GCaMP5G, UAS-TyrR2*^{RNAi}. (**D**) Knockdown of *TyrR1* instead of *TyrR2* or *Oct-TyrR* in ECs significantly increases intestinal neutral lipids. Three independent experiments were performed. *n* = 10 animals for each experiment. Two-way ANOVA analysis for statistics. Mean ± SEM was shown, **P* < 0.05, n.s. no significance. ORO intensity of R2 region was quantified for each condition. Genotypes: NP1Gal4, tubGal80^{ts}; *UAS-TyrR1*^{RNAi}, NP1Gal4, tubGal80^{ts}; *UAS-Oct-TyrR*^{RNAi}, or NP1Gal4, tubGal80^{ts}; *UAS-TyrR2*^{RNAi}. (**E**) Intestinal neutral lipids was examined by ORO staining when *TyrR1* was silenced in enteroendocrine cells by ProsperoGal4. Genotypes: ProsGal4, tubGal80^{ts}; *UAS-TyrR1*^{RNAi}. Biological triplicates were performed. Student t test for statistics, mean ± SEM was shown, n.s. no significance. (**F**) Related to Fig. 4H,I, CREB activities in ECs were quantified by fluorescence intensity of anti-p-CREB. Representative images were shown. Scale bar: 20μm. Genotypes: W^{1118} or CREB^{Δ36}, or NP1Gal4, tubGal80^{ts}; UAS-*Serca*^{RNAi}. (**G**) Related to Fig. 4L, CRTC overexpression suppressed HFD-induced intestinal lipid accumulation. Representative images of ORO staining in R2 region were shown. Genotypes: NP1Gal4, tubGal80^{ts} or NP1Gal4, tubGal80^{ts}; UAS-*Serca*^{RNAi}. Scale bar: 200 μm.

 © The Author(s)
